# Abdominal Adiposity and Physical Inactivity Are Positively Associated with Breast Cancer: A Case-Control Study

**DOI:** 10.1155/2018/4783710

**Published:** 2018-07-12

**Authors:** Jordana C. M. Godinho-Mota, Larissa V. Gonçalves, Leonardo R. Soares, João F. Mota, Karine A. Martins, Ismael Freitas-Junior, Ruffo Freitas-Junior

**Affiliations:** ^1^Department of Obstetrics and Gynecology, Federal University of Goiás, St. 227, Block 68, Setor Leste Universitário, 74.605-080 Goiania, GO, Brazil; ^2^Clinical and Sports Nutrition Research Laboratory (Labince), Faculty of Nutrition, Federal University of Goiás, St. 227, Block 68, Setor Leste Universitário, 74.605-080 Goiania, GO, Brazil; ^3^Physical Education Department of Julio de Mesquite Filho State University of São Paulo, Roberton Simonsen Ave, 19060-000 Presidente Prudente, SP, Brazil

## Abstract

**Objective:**

To examine whether breast cancer is associated with body composition and level of physical activity, considering the menstrual status.

**Methods:**

This was a case-control study with 116 women recently diagnosed with breast cancer and 226 controls. Body composition was assessed by dual-energy X-ray absorptiometry, and cardiometabolic risk was assessed by conicity index and waist-to-height ratio. The short version of the International Physical Activity Questionnaire was used to estimate the level of physical activity. All analyses were adjusted for age and BMI.

**Results:**

The total body fat percentage, android body fat, android-gynoid ratio, and waist circumference were positively associated (p < 0.05), whereas the percentage of lean body mass (p <0.05) and the level of physical activity (p < 0.01) were inversely associated with breast cancer in premenopausal women. Among postmenopausal women, physical activity decreased the chance of developing breast cancer by 49% (95% CI = 0.29 to 0.92, p = 0.02).

**Conclusion:**

A low percentage of lean body mass and high abdominal adiposity in the premenopausal period increase the chances of developing breast cancer. Regular physical activity is inversely associated with breast cancer in pre- and postmenopausal women.

## 1. Background

Breast cancer is the most common cancer among women in developed and developing countries [1, 2]. This type of cancer has a multifactorial etiology and diverse tumor biology, with increasing incidence in recent decades [1–3]. These epidemiological aspects associated with high mortality have motivated the investigation of the possible risk factors, especially the modifiable risk factors as diet, weight, physical activity level, and alcohol consumption [2–5].

In recent years, several studies have associated physical activity with a reduced risk of breast cancer, especially in women after menopause and those who have not been exposed to hormone replacement therapy [4–7]. However, a prospective study showed that exercise before menopause is associated with a 23% lower risk of premenopausal breast cancer [6]. According to Neilson et al. (2017) [4], high versus lower levels of moderate-vigorous activity were associated with lower risk of pre- and postmenopausal breast cancer. On the other hand, in overweight/obese women, the benefit from moderate-vigorous activity for breast cancer prevention may vary according to menopause status. Some studies [3, 8] considered obesity as a risk factor for breast cancer only in postmenopausal state. Obesity and body composition are also associated with breast cancer by increasing the levels of estrogen, proinflammatory cytokines, insulin resistance, and breast density [3, 8, 9].

Most epidemiological studies use body mass index (BMI) as an indirect measure of adiposity [3, 8]. Despite that, BMI shows individual heterogeneity, and it does not differentiate the body compartments or quantify the distribution of fat [3, 8–10]. Therefore, it is important to conduct research on effective methods to distinguish the body components.

It remains uncertain whether there is an association between breast cancer and body composition when analyses are adjusted by BMI and when considering the menstrual status [3, 8, 9, 11, 12]. A large between-study heterogeneity was observed in the application of diagnostic criteria and choice of the assessment of body composition components [10]. Therefore, our study aimed to examine whether breast cancer associates with body composition and level of physical activity even after controlling for the effects of BMI and age in pre- and postmenopausal women. We hypothesized that there is a positive association between abdominal adiposity, physical inactivity, and breast cancer.

## 2. Methods

### 2.1. Study Design and Sample

This was a case-control study conducted in a referral public hospital in the diagnosis and treatment of breast cancer. Data were collected from August 2014 to June 2016. Ethical approval was obtained from the Ethics Committee of the Federal University of Goiás (protocol number 751.387/2014). All participants were informed about the study orally and by writing and gave their written informed consent to participate.

For the sample size calculation, a standard deviation of ± 8.8 (%) for the total body fat variable [9] and a composition of two controls for each case were considered in order to find a statistical significance if the absolute value of the difference between the two groups was 4.0%. With a rejection power of the null hypothesis of 80%, a type I error of 0.05 (*α* = 5%), and a probable loss of 30%, a minimum sample of 75 cases and 150 controls was obtained, totaling 225 women.

The study included women aged between 30 and 80 years. In the case group, those with newly diagnosed breast cancer, prior to the start of chemotherapy, excluding carriers of metastatic disease or with a history of other cancers, were considered eligible. The control group included women who recently underwent mammography and/or physical examination of the breasts without changes and who had no history of breast cancer or other cancers. The presence of any cognitive impairment or psychiatric disorder, which would preclude the understanding of the work and the collection of the necessary information, and the presence of medical conditions that could compromise the nutritional status and/or harm physical activities were defined as exclusion criteria for both groups. Groups were matched for age, BMI, and menopause status.

### 2.2. Measurements

Initially, we conducted a pilot study for the adequacy, accuracy, and precision of the anthropometric and body composition measurements using a reference anthropometry standardization technique recommended by Habicht (1974) [13]. Sociodemographic data were collected to characterize the sample. Ethanol consumption (grams per day) was calculated according to the frequency, quantity, and type of alcoholic beverage ingested by the participant. Smoking status was determined based on the response to the question, “Do you now or have you ever smoked cigarettes, at least one a day for one year's time?”. If the answer was “yes”, the participant should report the average number of cigarettes smoked per day [14]. The level of physical activity was assessed using the International Physical Activity Questionnaire short form (IPAQ-SF) [15]. The IPAQ-SF assessed the sedentary time (sitting time) on a weekday during the previous week. The vigorous-intensity, moderate-intensity, and walking activity were multiplied by their estimated intensity in the metabolic equivalent (MET) and summed to gain an overall estimate of total physical activity per day [15]. The MET intensities used to score IPAQ were vigorous (8 METs), moderate (4 METs), and walking (3.3 METs) [15]. Women were classified as “physically inactive” if they had achieved less than 600 MET-minutes/week and “physically active” if they had reached at least 600 MET-minutes/week [16–18].

A digital scale with an accuracy of 0.1 kg and capacity of 150 kg was used for body weight (kg) evaluation. Height was measured with a stadiometer, with an accuracy of 0.1 cm, using a standard technique [19]. The waist circumference was measured with an inelastic tape placed at the midpoint between the anterior superior iliac crest and the last rib. Subsequently, the patients were classified as at risk of metabolic complications using ≥ 80 cm as a cut-off point [20]. The following ratios were obtained from these anthropometric measurements: BMI, calculated by the ratio between weight and height squared (kg/m^2^); the waist-height ratio (WHtR), obtained by dividing the waist circumference (cm) by height (cm) [21]; and conicity index, calculated according to Valdez (1991) [22].

Body composition was determined using dual-energy X-ray absorptiometry with a GE Lunar densitometer (DPX NTVR, GE) with the enCORE 2011 software (version 13.60, GE Healthcare). All metal objects were removed from the women before the scan. The tests included a complete body scan of the patients, in supine position, and all the measures and calibrations were performed by the same operator [23]. At the end of the evaluation, we collected the values in percentage of lean body mass (LBM), total body fat, gynoid and android fat, and android-gynoid fat ratio. The performance of the equipment was evaluated by calibration block on a daily basis and by spine phantom on a weekly basis. The coefficients of variation for the tests of muscle and fat mass were 0.75% and 1.03%, respectively.

### 2.3. Statistical Analysis

The database was entered in duplicate in the Epi-Info™ software (version 7.1.5), and data analysis was conducted using Stata software for Windows (version 12.0), considering the outcome diagnosis of breast cancer. We used the Shapiro Wilk test to verify the distribution of continuous variables. The chi-square test was used to identify possible differences in demographic, clinical, and behavioral characteristics between groups. Associations between body composition and physical activity were examined using Pearson's or Spearman's correlation coefficient for parametric and nonparametric samples, respectively. We applied the Mann–Whitney U test (nonparametric distribution) to compare continuous variables between groups. Subsequently, age- and BMI-adjusted logistic regression analysis were determined in order to get the odds ratio (OR) and 95% confidence interval (95% CI). Variables with a significance level of less than 0.05 were considered to be associated with breast cancer.

## 3. Results

A total of 342 women were included in the study: 116 cases and 226 controls. There was a predominance of non-Caucasian (70.00%) and postmenopausal women (61.11%). Sixty-seven percent of the women were overweight according to their BMI (n = 232), and this prevalence was higher when assessed by DXA (95.61%, n = 327). The mean age ± standard deviation was 41.88 ± 6.61 and 42.40 ± 6.40 years among the premenopausal women and 58.80 ± 8.07 and 59.17 ± 7.74 years among the postmenopausal women for cases and control, respectively. Age, ethnicity, menopause status, BMI, percentage of smokers, and alcohol consumption did not differ among groups; however, the level of physical activity was higher in control when compared to cases (Table 1). BMI (r = 0.02, p = 0.71) and body weight (r = 0.02, p = 0.62) were not associated with MET. However, waist circumference (r = -0.14, p = 0.01), % body fat mass (r = -0.17, p = 0.002), % android fat (r = -0.16, p = 0.003), conicity index (r = -0.15, p = 0.007), and WHtR (r = -0.11, p = 0.04) were negatively associated with physical activity, while LBM percentage (% LBM) (r = 0.16. p = 0.002) was positively associated.

**Figure 1:**
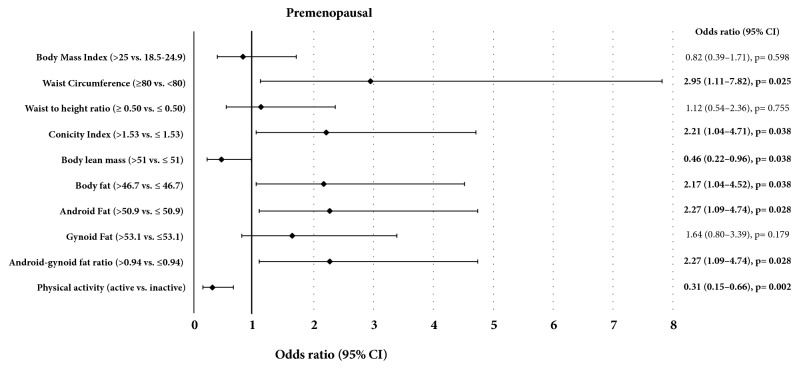
Association of body composition, physical activity, and breast cancer risk in women according to premenopausal status.

**Figure 2:**
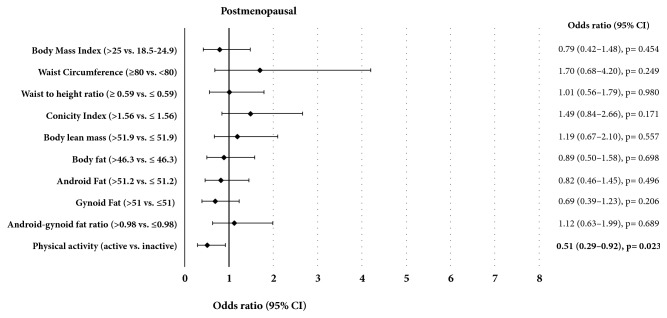
Association of body composition, physical activity, and breast cancer risk in women according to postmenopausal status.

Premenopausal women with breast cancer had a higher conicity index (p = 0.003) and lower levels of physical activity (p < 0.001) when compared to the control group (Table 2). The % LBM 0.46 (0.22 to 0.96, p = 0.038) and the physical activity status 0.31 (0.15 to 0.66, p = 0.002) were inversely associated with breast cancer in premenopausal women (Figure 1). On the other hand, the total body fat, percentage of android fat, android-gynoid fat ratio, waist circumference, and conicity index were positively associated with breast cancer in the same group (p < 0.05, Figure 1). In postmenopausal women, the physical activity status was also inversely associated with the risk of developing breast cancer, representing a reduction of 49% in that risk (Figure 2).

## 4. Discussion

Our study showed that physical inactivity, excessive total and abdominal body fat, and lower % LBM are associated with breast cancer in premenopausal women, whereas in postmenopausal women only physical inactivity is associated with the outcome. Some studies have linked breast cancer to these factors. However, in subgroup analysis according to the menstrual status, the results remain controversial. Among the factors that could explain the different results for the association between physical activity, body composition, and risk of breast cancer are the different levels of physical activity [4, 5] and methods used to assess nutritional status [3, 5, 8, 9, 11, 12].

According to Howell et al. (2014) [5], there are still major gaps concerning risk assessment and prevention of breast cancer, and new biomarkers for risk prediction are likely to come from measures in tissues by a variety of techniques. BMI is a commonly used method due to its simplicity of measurement, low cost, and high reproducibility [8]. The last Expert Report published in 2010 by the American Institute for Cancer Research refers mainly to studies using this method as a measure of total body adiposity [24]. This was also observed in the review conducted by Howell et al. (2014) [5]. However, BMI may not represent a direct association with body composition. Therefore, some eutrophic women may hide a reduced muscular mass under an apparently normal adipose mass, perhaps reproducing a situation similar to “sarcopenic obesity” [10], which could be associated with a higher risk of breast cancer. Moreover, some overweight women could present normal body fat mass associated with high % LBM, thus showing more adequate proportions of both components. Therefore, these factors may not be associated with breast cancer risk [9, 11, 12]. Furthermore, in our study we did not verify an association between BMI and physical activity, whereas adiposity markers were negatively associated with physical activity, and %LBM showed a positive correlation. These results suggest the importance of analyzing body composition comprehensively.

One of the possible factors that could associate obesity to a probable protection against breast cancer in the premenopausal stage would be the anovulatory effect which reduces the cumulative exposure of the breast to estrogen. However, this physiological change usually occurs with BMIs consistent with severe obesity [25, 26], and the prevalence of this situation in our study was less than 5%, thus reducing that possible protective effect. Moreover, the association between abdominal fat and breast cancer in premenopausal women may be more related to higher production of insulin, insulin growth factor 1, and adipokines than to estrogen [4, 6, 26].

A cohort study conducted in the United States, with a mean follow-up of 12.9 years, observed that postmenopausal women with a higher percentage of body fat and fat accumulation in the trunk, both measured by DXA, had a higher risk for developing breast cancer (OR 1.60, 95% CI 1.18 to 2.18; OR 2.05, 95% CI = 1.50 to 2.79, respectively) [3]. The differences between studies may be due to the fact that most women in the American study were white and had a lower level of physical activity compared to the women in our study [3]. In addition, the American study did not follow premenopausal women, a limiting factor considered by the authors [3].

The relevance of LBM as a protective factor for breast cancer has been questioned [11]. The highest % LBM is corroborated with a lower percentage of body fat and thus with lower estrogen production through aromatization of androgens and the reduction of proinflammatory cytokines secretion [3, 11, 12]. Immune inflammatory cells can be actively tumor-promoting, given that they are capable of fostering angiogenesis, cancer cell proliferation, and invasiveness [27]. Further, through a feedback mechanism, cytokines can stimulate muscle degradation and inhibit protein synthesis, thus reducing the % LBM [10, 11, 28].

A case-control study with 343 Uruguayan women newly diagnosed with breast cancer and 1,125 healthy controls homogenized by age was conducted in a public hospital. The authors observed a positive association between the lowest % LBM and a higher percentage of body fat with breast cancer. The heterogeneity between premenopausal and postmenopausal women was analyzed by means of the likelihood ratio test, but there was no effect modification from menopausal status. The authors suggest that muscles can improve insulin sensitivity by regulating the immune system and myokine production, reducing inflammatory status [11].

No association was found between BMI and breast cancer in women of African ancestry [12]. On the other hand, an increase of 5 cm in waist circumference, adjusted for BMI, increased the risk for breast cancer by 10% in premenopausal women (OR = 1.10; 95% CI: 1.00 to 1.22). Abdominal adiposity increases insulin, insulin growth factor 1, androgens, estrogen, and leptin, and it reduces the production of adiponectin and sex hormone-binding globulin (SHBG) in black women before the onset of menopause [3, 12]. According to Paxton et al. (2013) [29], waist circumference is associated with higher levels of estradiol and SHBG in women of African ancestry. Most women in our study were also of African ancestry, which may explain the similarities between the results.

The practice of physical exercises has been highlighted as an important protective factor against breast cancer, since it reduces serum levels of estrogen, insulin, leptin, and proinflammatory cytokines and increases adiponectin. Besides contributing to a lower body adiposity [4, 6], exercise may also contribute to epigenetic regulation of breast cancer enhancing tumor-suppressing genes expression or reducing oncogenes expression [30, 31] and increasing production of anti-inflammatory proteins that may well contribute to an antitumor activity [32]. On the other hand, moderate-vigorous recreational activity in overweight and obese menopausal women was not associated with breast cancer prevention. Different from intense and purposeful activity (e.g., exercise), recreational activities reflect an overall lifestyle which provide little benefit for breast cancer prevention [4]. However, clarifying the physiological mechanisms of physical activity in the protection against breast cancer is still a challenge, in addition to the difficulty of quantifying the intensity and duration accurately [7].

Friedenreich et al. (2015) verified a reduction of approximately 25% in the risk of breast cancer in active versus inactive women in different population groups and all types of activities (leisure, occupational, or domestic) [7]. These findings probably relate to the better metabolic control and lower adiposity associated with physical exercise practice. In our study, after logistic regression analysis adjusted for age and BMI, we found that both pre- and postmenopausal active women had a lower risk of breast cancer (Figures 1 and 2).

Some potential limitations of our study were the case-control study design, the use of a questionnaire to assess the level of physical activity, and the absence of food consumption analysis.

In conclusion, we found that a low % LBM and a high abdominal adiposity in premenopausal women were associated with breast cancer. In addition, high level of physical activity remained inversely associated with breast cancer in pre- and postmenopausal women.

## Figures and Tables

**Table 1:** Demographic, clinical, and behavioral characteristics among participants.

Variables	Controls (n = 226)	Cases (n = 116)
Age (years)*∗*	52.8 ± 11.2	52.8 ± 11.8
Race/ Ethnicity		
Caucasian (%)	30.0	30.2
Non-Caucasian (%)	70.0	69.8
Menopause status		
Premenopausal (%)	38.9	38.8
Postmenopausal (%)	61.1	61.2
BMI (kg/m^2^)*∗*	28.1 ± 4.9	28.4 ± 5.2
Smoking status (cigarettes/ day)	1.5 ± 5.8	1.2 ± 5.5
Ethanol intake (g/ day)	1.2 ± 3.8	1.5 ± 4.3
MET (min/week)*∗*^†^	1663 ± 2509	1186 ± 1914

*∗* Nonparametric variables (Shapiro-Wilk test).

^†^ p < 0.01 controls vs. cases (Mann–Whitney U test).

**Table 2:** Differences in body composition and level of physical activity between cases and controls according to menopausal status.

	Premenopausal			Postmenopausal		
Variables	Cases (n = 45)	Controls (n = 88)	p*¹*	Cases (n = 71)	Controls (n = 138)	p*¹*
Body weight (kg)*∗*	70.0 ± 13.6	69.4 ± 12.9	0.956	67.9 ± 12.0	68.5 ± 12.7	0.636
Height (m)	1.6 ± 0.06	1.6 ± 0.06	1.000	1.6 ± 0.05	1.6 ± 0.06	0.261
BMI (kg/m^2^)*∗*	28.1 ± 5.2	27.7 ± 5.3	0.618	28.5 ± 5.2	28.3 ± 4.7	0.759
Waist circumference (cm)	91.9 ± 12.4	87.4 ± 13.2	0.058	93.1 ± 11.2	92.2 ± 11.9	0.780
Waist–height ratio	0.57 ± 0.08	0.55 ± 0.09	0.092	0.60 ± 0.07	0.58 ± 0.07	0.515
Conicity index	1.5 ± 0.11	1.4 ± 0.12	**0.003**	1.5 ± 0.10	1.5 ± 0.10	0.131
Body fat (%)*∗*	45.7 ± 6.5	44.0 ± 6.7	0.103	45.2 ± 6.5	45.9 ± 6.1	0.322
Body lean mass (kg)*∗*	36.2 ± 5.9	37.4 ± 6.7	0.070	35.4 ± 4.2	35.2 ± 5.2	0.326
Android fat (%)*∗*	48.6 ± 8.2	46.3 ± 9.1	0.111	49.9 ± 6.8	50.4 ± 7.1	0.381
Gynoid fat (%)	52.3 ± 5.8	51.5 ± 5.1	0.338	50.9 ± 5.7	52.1 ± 5.4	0.170
Android–gynoid fat ratio*∗*	0.92 ± 0.11	0.89 ± 0.15	0.200	0.98 ± 0.10	0.96 ± 0.10	0.498
MET (min/week)*∗*	1120 ± 2274	1754 ± 2348	**<0.001**	1227 ± 1662	1605 ± 2614	0.157

*∗* Nonparametric variables (Shapiro-Wilk test).

^†^ p < 0.01 controls vs. cases (Mann–Whitney U test).

## Data Availability

The data used to support the findings of this study are available from the corresponding author upon request.

## Abbreviations

BMI: Body mass index
IPAQ: International Physical Activity Questionnaire
MET: Metabolic equivalent
WHtR: Waist-height ratio
LBM: Lean body mass
% LBM: Percentage of lean body mass
OR: Odds ratio
DXA: Dual-energy X-ray absorptiometry
SHBG: Sex hormone-binding globulin.
